# Microbes on Clipper Blades after Use and Disinfection in Small Animal- and Equine Practice

**DOI:** 10.3390/vetsci11010038

**Published:** 2024-01-17

**Authors:** Lina Gustafsson, Camilla Wikström, Ralf S. Mueller, Kerstin Bergvall

**Affiliations:** 1 Evidensia Södra Djursjukhuset, Månskärsvägen 13, 141 75 Huddinge, Sweden; kerstin.bergvall@evidensia.se; 2 Swedish Veterinary Agency, 751 89 Uppsala, Sweden; 3 Centre for Clinical Veterinary Medicine, LMU Munich, Veterinärstrasse 13, 80539 Munich, Germany; 4 Department of Small Animal Clinical Sciences, Faculty of Veterinary Medicine and Animal Science Swedish, University of Agricultural Sciences, P.O. Box 7070, 750 07 Uppsala, Sweden

**Keywords:** hygiene routines, clippers, disinfection, dermatophytes, sterilization, equine, feline, canine

## Abstract

**Simple Summary:**

Clipping hair on animals is necessary for many reasons, such as shaving the fur before an ultrasound or during catheter placement. There is a risk that bacteria from the skin contaminate the clipper blade, and potentially, are then transferred to another individual. This study aimed to investigate if the disinfection routines used in three small animal hospitals and one equine department in Sweden succeeded in removing bacteria and dermatophytes from clipper blades. The results indicate that the current disinfection routines are not enough to remove bacteria from used clipper blades, and that sterilization would be a more reliable way to minimize the risk of contamination.

**Abstract:**

Clipping hair on animals can produce microtraumas of the skin and the dislodgement of microorganisms to the clipper blade. This study evaluates if clipper blades in animal hospitals in Sweden are contaminated with bacteria and/or dermatophytes after disinfection. Eleven clipper blades from three veterinary referral hospitals, including one with a small animal department and an equine department, were sampled for bacteria and dermatophytes. All the hospitals had disinfection routines in accordance with the national recommendations for hygiene in veterinary medicine. The sampled clipper blades were supposed to be disinfected and they were considered to be ready for use by staff. Five sterilized clipper blades were used as controls. The results showed that 64–100% of the disinfected clipper blades, from all three hospitals, were contaminated with bacteria, whereas all the sterilized clipper blades were negative for bacterial growth (*p* < 0.05). One clipper blade from the equine department was contaminated with dermatophytes. The results indicate that the disinfection routines were not sufficient for removing bacteria from used clipper blades, and that sterilization would be a more reliable way to minimize the risk of contamination.

## 1. Introduction

Clippers are used daily in animal healthcare. Clipping hair on animals produces a risk of microtraumas of the skin [1] and the dislodgement of microorganisms from the skin surface and hair follicles to the clipper blade. This could pose a risk of infection to the next patient, particularly if invasive procedures such as surgery or catheter placement follow. If hand hygiene among staff is insufficient, which could be the case despite education campaigns [2], the clipper could also serve as a fomite for skin-related, human microorganisms.

With the increasing prevalence of antimicrobial resistance, it is of utmost importance to prevent bacterial infections, and to establish hygiene standards in animal healthcare that correspond with that goal. Nosocomial infections are often caused by opportunistic pathogens [3] and many of these can survive for months in the environment [4]. Several studies have found bacterial contamination on surfaces and materials [5,6,7] used in veterinary healthcare. Few studies have specifically investigated bacterial contamination on clipper blades. Furthermore, to the authors’ knowledge, no study has investigated microbial contamination of clipper blades in equine health care units.

In addition to bacterial contamination, fungal elements might contaminate the tools. Dermatophytosis is a fungal skin disease that can affect both animals (including cats, horses, and dogs) and humans. The zoophilic dermatophytes have their main niche in animals, but some geophilic species may also affect companion animals, even if soil is their main habitat. In dogs and cats, *Microsporum canis* is the most common dermatophyte [8]. In horses, the most prevalent species worldwide is *Trichophyton equinum* [9]. As animals can be carriers of dermatophytes without clinical signs [10], personnel might not always consider dermatophytosis when shaving. This could potentially lead to the unintended spread of arthrospores, although the clinical relevance is not yet known [11]. The zoonotic dermatophyte, *Microsporum canis*, was cultured from 30% of 50 sampled veterinary floors in Italy [12]. In another study, 235 pieces of equipment were sampled from groomers and veterinary clinics in Turkey. *Microsporum canis* was isolated from three blades and a toothbrush, and *Trichophyton tonsurans* was isolated from two clipper blades [13]. Even though dermatophyte infections from the environment is uncommon, microtrauma of the skin (due to, for example, clipping hair) could enhance the risk for infection in immunocompromised individuals [8].

Attempts have been made to find the optimal disinfectant solution and cleaning protocol for clippers. In a clinical situation, many factors can influence efficacy. In one study, different cleaning protocols for clipper blades were compared. Out of 60 sampled clipper blades from 60 clinics in the United States, bacteria grew on 51% of them. There was no significant association between contamination and storage location, cleaning frequency, or type of veterinary practice. Similarly, there was no association between contamination and a specific disinfection agent. The authors were unable to determine which protocol for disinfection should be recommended [14]. In another study, sterile clipper blades were inoculated with *Pseudomonas aeruginosa*, *Staphylococcus (S.) aureus*, and *Escherichia coli*; then, they were sprayed or soaked in disinfecting solutions for 20 min. Three of the five detergents—ethanol/o-phenylphenol spray, isopropyl alcohol, and chlorhexidine soaks—were successful in eradicating bacteria [15]. However, the situation in a clinical setting is different, as hair and debris accumulate, and the cleaning technique among clinics and staff could differ.

When clipper blades from grooming salons were sampled in USA, both methicillin-resistant *S. aureus* (MRSA) and methicillin-resistant *S. pseudintermedius* (MRSP) could be isolated [16]. MRSA, and especially MRSP, have emerged as important threats in veterinary medicine in recent decades. Limited treatment options for those bacterial infections cause increased morbidity and mortality. Methicillin-resistant *S. aureus* is of huge importance in human healthcare, but it is also widespread outside of hospitals. The strains associated with dogs and cats usually come from humans [17], but the transmission route can also be reversed [18]. For a long time, Nordic countries advocated the prudent use of antimicrobials to minimize the development of multidrug resistance. The prevention of infections is one of the fundamental principles in this work. The Swedish Veterinary Association makes recommendations for the disinfection of clippers in the national guidelines for infection control in animal health care. In the recommendations for small animal veterinary health care, it is stated that the clipper machine should be wiped with an alcohol-based surface disinfectant, containing surfactant, after every patient. The blade should be removed and wiped in the same way or washed with a dish disinfector [19]. The recommendations for equine health care are similar [20]. The prerequisite for any routine to be efficient is that the instructions are adequate and that they are followed by staff. The aim of this study was to evaluate if the disinfection routines, in accordance with the national recommendations, are sufficient to remove the bacterial and/or dermatophyte contamination of clipper blades in small animal and equine health care. Sterilized clipper blades were used as controls. As we wanted to determine contamination levels in a real clinical situation, we sampled the clipper blades after they were supposed to be disinfected for use again shortly afterwards.

## 2. Materials and Methods

### 2.1. Study Design

This was an open-label, non-randomized study. Three hospitals (H1, H2, H3) in Sweden, with different owners, were included. All the hospitals have a busy emergency department, wards, and specialist clinics. One of them has both a small animal- and a horse unit. The participating hospitals had similar, yet slightly different protocols for disinfection of clipper blades (Appendix A). All of the instructions prescribed cleaning the clipper blades with a toothbrush to remove debris and hair. In hospital one (H1), the blades were treated with antibacterial wipes containing 45% isopropanol. In hospital two (H2), a mixture of 40% isopropanol and 10% ethanol was poured onto the blades in the small animal department. In the equine clinic, a spray with 45 % isopropanol was sprayed on the blades. In hospital three (H3), a 40% isopropanol solution was poured onto the blades. Sometimes, 75% ethanol was used instead.

Eleven cleaned and disinfected clipper blades were sampled in each hospital, except for H2, where eleven blades were sampled in the small animal department, and eleven were sampled in the equine department, respectively. Five autoclaved clipper blades from H1′s dermatology department for small animals were used as controls. The staff in the clinics were not informed of the study, nor given notice of the sampling beforehand. The sampling occurred unannounced, and the disinfection procedures followed ordinary routines. The sample size was determined on the basis of results from a pilot study, and a power of 80%. A smaller number of autoclaved blades was used as they were less likely to differ from one another.

### 2.2. Sample Collection for Bacteria

Clipper blades that were expected to have been disinfected, considered by the staff to be ready to use, were sampled. The clipper machines were of two different sizes (small/large), and they had different trademarks; the most commonly used were Aesculap Isis for small clippers and Oster for the large ones. In both variants, the blade could be detached from the clipper machine. An assistant held the clipper handle so that the blade did not touch anything, and in cases when the blade was detached from the machine, they held the blade with sterile gloves that were changed between each sampling. An e-swab with a ‘minitip’ was used for bacterial sampling. The tip was dipped into the transport medium before it was swabbed between the teeth of the blade, as well as above and under the blade.

### 2.3. Sample Collection for Fungi

A clean toothbrush, and a dry, sterile, minitip were swiped between the teeth of the blade, and above and under the blade, for dermatophyte culturing. Then, they were stored in a dry, airy package for transport.

### 2.4. Storage of Clippers and Blades

In H1, the nurses have their own personal, clippers which are stored on a specific shelf in a corridor where staff and animals pass by. The clippers were sampled in the afternoon, when they were placed in their chargers on the shelf, with the blade pointing upwards; they did not directly come into contact with anything in the environment.

In H2′s small animal and equine departments, the sampling occurred in the morning. Some clipper blades were collected from visibly clean, open steel containers, which were designated for drying disinfected clipper blades that had been detached from the machine. The steel containers were placed in areas where staff and animals pass throughout the day. Other clippers were collected from individual nurses when they considered them clean. Some were taken from charger stations in rooms where clippers were used, for example, from the room where abdominal ultrasounds take place. In the charger stations, the clippers are positioned so the blades point upwards. 

In H3, the sampling was conducted in the afternoon. Clippers were collected from charger stations in rooms for abdominal and cardiac ultrasounds, where they were expected to have been disinfected. Some were collected from individual nurses who considered them clean after disinfection.

Clippers that belonged to, and were used in the surgery departments, were not sampled and part of the study in any hospital. 

### 2.5. Bacterial Cultures

The samples were transported to the laboratory at the Swedish Veterinary Agency on the same day, where they were cultured on bovine blood agar plates and bromocresol purpur lactose agar plates. They were incubated at 37 °C and checked after 24 and 48 h. In the absence of visible growths on the primary plates after 24 h, the original sample was inoculated in bovine serum broth with 10% horse serum, for non-selective enrichment. It was incubated at 37 °C, and checked after 24 and 48 h. If the content was turbid after 24 h, 10 μL of the enrichment broth was streaked on agar plates. Otherwise, this was conducted regardless of turbidity after 48 h. The plates were then incubated for 24 and 48 h before bacterial growth was assessed.

### 2.6. Fungal Cultures

Fungi were cultured for 15 days. The toothbrushes were carefully pressed onto modified Dixon agar. Some straws were cut from the toothbrush onto the same plate. New sterile scissors were used for every sample. The dry minitip was cultured on Sabouraud agar. The plates were incubated at 27 °C (±2 °C) and read every day during the first week; then, they were read twice weekly. If there was suspected growth of *Microsporum canis*, the isolate was re-cultured on a rice medium for species identification. Colonies on the agar plates were read macroscopically, and suspected colonies were studied microscopically after sampling the culture surface with a tape. The tape technique is a method used to quickly define the species of dermatophytes. The sample was studied using a light microscope at 10× or 40× magnification. The species were identified by determining the morphology of the hyphae, spores, conidiogenous cells, and conidia.

### 2.7. Typing of Bacteria

The bacteria were typed with a MALDI Biotyper (MALDI-TOF). A score between 2.3 and 3 means highly probable species identification. The lower the score, the less secure the species identification. If the score was <2, a sample was re-run after purification to obtain fresh material. If that gave a result with a MALDI-TOF between 1.7 and 2, the bacteria were assessed on a genus level. For scores under 1.7, bacteria were assessed after Gram staining.

### 2.8. Quantification of Bacteria

If any bacterial growth was present, cultures were classified as contaminated. Colonies were counted and the growth was quantified in accordance with the following definition: single colonies, 1–3 colony forming units (CFU); sparse growth, <10 CFU; moderate growth, 10–50 CFU; and rich growth, >50 CFU.

### 2.9. Statistical Analysis

A comparison between the clipper blades with bacterial contamination, the controls, and the individual hospitals, was conducted using Fisher’s exact test (Prism 9.5.0, Graphpad, San Diego, CA, USA). A *p*-value of <0.05 was considered significant.

## 3. Results

### 3.1. Bacterial Contamination

**Figure 1:**
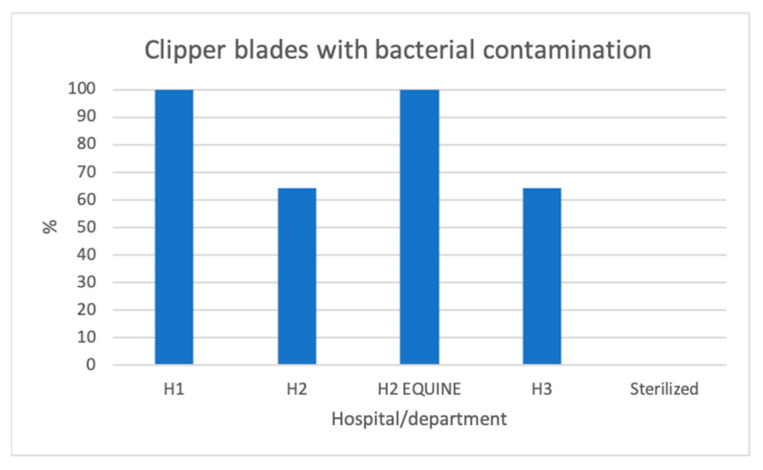
Percentage of clipper blades with bacterial contamination. H1 = hospital 1; H2 = Hospital 2; H2 Equine = Equine department in hospital 2; Sterilized = the autoclaved clipper blades from hospital 1.

**Figure 2:**
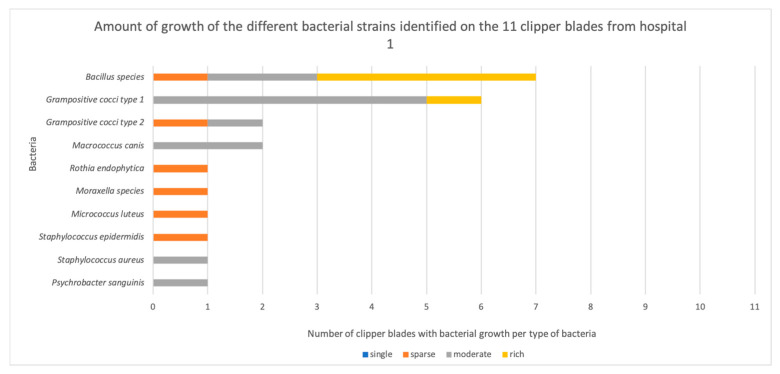
The amount of growth of the different bacterial strains identified on the 11 clipper blades from hospital 1.

**Figure 3:**
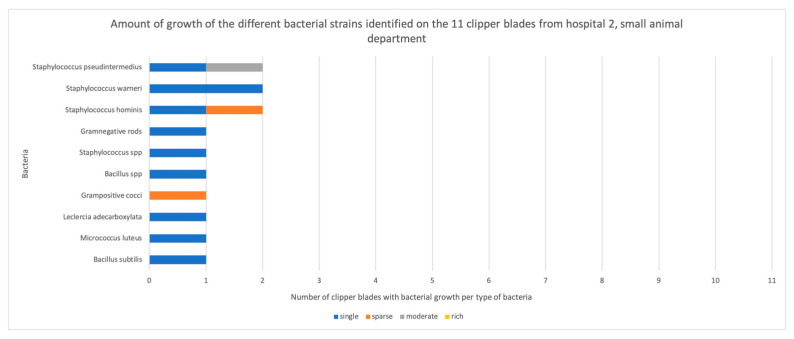
The amount of growth of the different bacterial strains identified on the 11 clipper blades from the small animal department in hospital 2, before enrichment.

**Figure 4:**
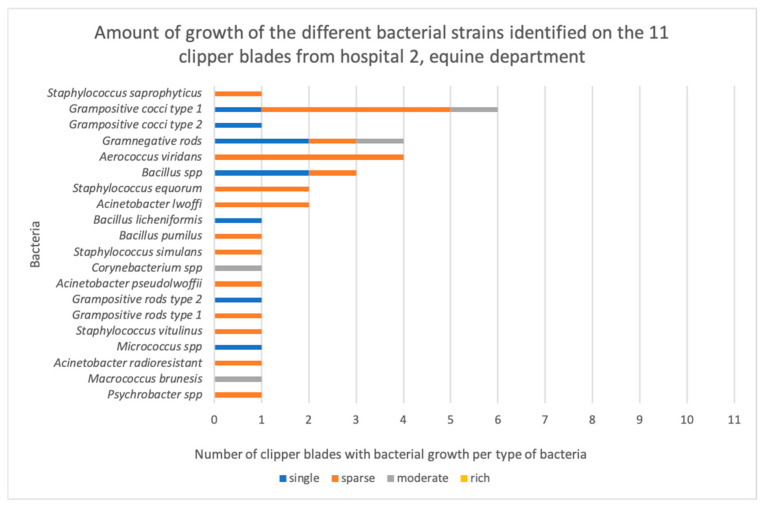
The amount of growth of the different bacterial strains identified on the 11 clipper blades from the equine department in hospital 2.

**Figure 5:**
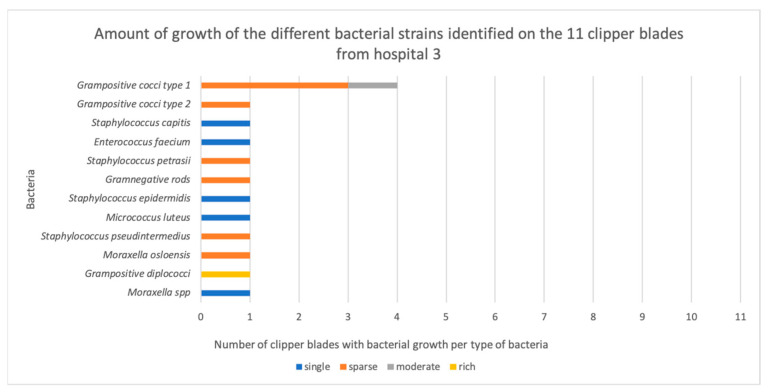
The amount of growth of the different bacterial strains identified on the 11 clipper blades from hospital 3, before enrichment.

Out of 44 disinfected, but not sterilized, clipper blades, bacteria grew on 36 blades (88%, Appendix B). More than one bacterium grew on 29 of them. Bacterial growth occurred on 100% (11/11) of the blades from H1, compared with none of the autoclaved control blades. This was a highly significant difference (*p* = 0.0002). Similarly, growth occurred on 64% (7/11) of the blades from H2′s small animal department (*p* = 0.0337), 100% (11/11) of the blades from H2′s horse clinic (*p* = 0.0002), and 64% (7/11) of the blades from H3 (*p* = 0.037) (Figure 1). There was no significant difference between the hospitals (*p* = 0.902) in terms of the number of clippers with bacterial contamination. However, six out of seven contaminated clipper blades in H2′s small animal department exhibited single colony growth, and six out of seven in H3 exhibited single colonies or sparse growth, whereas in H1, all the blades exhibited either sparse, moderate, or rich growth of bacteria (Figure 2, Figure 3, Figure 4 and Figure 5).

After enrichment in a serum broth, and another 24 h of incubation, four out of four originally negative cultures from H2′s small animal department were positive. None of the four negative clipper blades in H3 exhibited growth after enrichment. Cultures of the sterilized clipper blades after enrichment were also negative.

### 3.2. Fungal Contamination

One clipper from the equine department in H2 exhibited growth of *Trichophyton*, suspected *terrestre*. The identification of the subspecies could not be established, but *Trichophyton mentagrophytes* or *Trichophyton equinum*, which are the more common *Trichophyton* species associated with infection in dogs, cats, and horses, could be excluded. *Malassezia pachydermatis* grew on one clipper blade from H1. A statistical comparison between the blades infected with fungi and the control blades was not sensible due to the small number of blades contaminated with fungi.

## 4. Discussion

There was a high prevalence of bacterial contamination on clipper blades from all three hospitals in the study, and from both the small animal departments and the equine clinic. Different species were present in different amounts (Appendix B).

Cutaneous bacteria such as *S. pseudintermedius* grew in moderate amounts on one clipper blade from H2′s small animal department, in single colonies on another blade from H2′s small animal department, and in sparse amounts on one blade from H3. *S. aureus* grew in moderate amounts on one clipper blade from H1.

*S. pseudintermedius* and *S. aureus* are coagulase-positive facultative anaerobic staphylococci and two of the most relevant opportunistic pathogens in veterinary medicine [21]. They are part of the normal flora of the skin and mucous membranes in dogs and cats [22]. *S. pseudintermedius* is the most common pathogen in canine superficial pyoderma [23,24], and *S. aureus* is the most common pathogen in cats with inflammatory skin disease [22] and pyoderma [25]. In the current study, we did not test for methicillin resistance, as the prevalence in Sweden is low and this was not the focus of the study. However, the fact that bacteria is present on disinfected clippers indicates that there is a potential risk for transmission of MRSA and MRSP, regardless of whether these specific colonies carried the gene for methicillin-resistance or not.

Among other bacteria on the clipper blades were coagulase-negative staphylococci, which are commensals on the skin and mucous membranes of humans, dogs, cats, and horses [26]. They are not considered primary pathogens, and the risk of causing a skin infection in a healthy individual is low. However, in immunosuppressed individuals they can be a potential risk, and they may also enter via joint or contaminate implants during orthopedic surgery [26]. In human medicine, the most common nosocomial infection is caused by coagulase-negative *S. epidermidis*, which is often associated with the colonization of implants [27,28]. As with many other coagulase-negative staphylococci, the main virulence factor is the ability to form biofilm [27], which enhance their ability to colonize surfaces. They can also carry genes for antimicrobial resistance, which can be transferred to bacteria with more pathogenic potential [29]. The clinical significance of this is not fully elucidated. 

Many clippers were contaminated with *Bacillus* species. *Bacillus* are spore-forming environmental bacteria with an ability to create biofilm. They are not likely to cause a primary cutaneous infection, but rare outbreaks have been reported in human medicine [30]. Many disinfectant solutions are unable to eradicate *Bacillus* spores completely [31] and some presence of *Bacillus* could therefore be anticipated with current routines. However, for the purposes of conducting a thorough investigation, it was considered relevant to report, as a protocol that results in as little contamination as possible should have the lowest risk of transmitting microorganisms.

Not all isolates could be identified by MALDI-TOF, which is likely due to the fact that it is primarily made for human pathogens. Another way to determine the species is via DNA sequencing, but as the main purpose of the study was to compare the presence of bacteria on sterilized versus disinfected clipper blades, this was not prioritized.

There was no growth of clinically relevant dermatophytes; nevertheless, a *Trichophyton* species grew on one clipper blade in H2′s equine clinic. This indicates that zoophilic dermatophytes could also survive the disinfection routine. However, the result is difficult to interpret, as the prevalence of dermatophytosis is not reported in either small animal or horse practices in Sweden, to the best of the authors’ knowledge. It has also not been possible to retrieve reliable data from the hospitals participating in the study, partly due to obstacles concerning the record system and the fact that carriers without clinical signs would not be identified and recorded. Studies from other parts of the world on the prevalence of dermatophytosis have yielded different results depending on the location, climate, temperature, and if the animals are housed together (i.e., in catteries) or alone [32]. Findings in asymptomatic cats also vary widely, with numbers ranging from 0% to 27% in healthy pet cats, and 0% to 88% in stray cats [32]. In a recent study where 138 cats with and without skin lesions were sampled for fungal culturing, 20.29% were positive for *Microsporum canis*. Of these, 13.04% had skin lesions and 7.25% were asymptomatic [33]. That study was conducted in Thailand, where a humid and warm climate is favorable for dermatophyte growth. In Sweden, the climate is generally dry, and the temperature is much lower. When asymptomatic cats were sampled in England, which has a climate that resembles that of Sweden, 2.16% of 169 cats tested positive for *Microsporum canis,* the same with *T. mentagrophytes* [34].

Of the dermatologic conditions reported in 900 horses over the period from 1979 to 2000, dermatophytosis was the second most common diagnosis after bacterial folliculitis. It represented 8.89% of the 900 cases examined at the College of Veterinary Medicine at Cornell University [9].

The sparse contamination of the clipper blades by dermatophytes in the Swedish veterinary hospitals has various different potential explanations. It may be that the prevalence of dermatophytosis among animals in Sweden is in fact low. Another possibility is that the protocol was actually efficient in eradicating spores, with one exception that could have been due to a poorly performed procedure. The sampling technique could also have been insufficient. Dermatophytes live on hair or skin, and they need keratinous material to multiply. Even though some of the clippers had visible debris in the form of hair, most of them did not, which could make clippers a less favorable environment for dermatophytes. According to Moriello, removing spores via decontamination procedures is not difficult, as long as the surface can be washed [8]. Thus, a limitation of this study concerns the fact that the prevalence of dermatophytosis is unknown, and the sampling was conducted just once in every place. If exposure occurred a long time ago, the clipper could present negative results, regardless of the disinfection protocol. To assess the risk for the transmission of dermatophytosis more reliably, this should be investigated further. It would be sensible to clip animals with known dermatophytosis, disinfect the blades, and then sample them for culturing.

This study has more limitations. Bacteria could have been transferred to the clipper blades during sampling (from gloves/hands, or from the surrounding surfaces), even though precautions were taken to make the procedure as sterile as possible. The clipper, or clipper blades, were collected from storage facilities that were assigned for cleaned clippers, and they were ready to use. However, it was not controlled to ensure that every blade had actually been cleaned, or that the implementation of the disinfection procedure was correct. They could also have been exposed to air-borne pathogens as most of them were placed in locations where animals and staff pass. As the aim of the study was to evaluate whether the routines for disinfecting clippers were sufficiently able to remove microbial contamination in practice, we wanted to mirror unbiased, clinical situations. Therefore, the specific disinfecting solutions, storage locations, and performances of staff were not evaluated in this study. Focus was neither on the type of bacteria, nor the prevalence of multidrug-resistance. Rather, the aim was to assess the routines for disinfecting clippers as a whole, in practical, clinical settings, and to compare it with autoclavation—this is a process with few interpersonal operational differences, and it is very straightforward. It is easy to determine when a blade has been sterilized and when it has not, as they are packed in sealed envelopes in the autoclave. Conversely, disinfection performances in a busy, standard hospital could differ greatly, and dirty blades could be mistaken for being clean.

The high prevalence of contaminated clipper blades may be due to a variety of reasons. Cleaning routines may be too complicated or time-consuming to be carried out correctly in a clinical setting. Alternatively, the disinfectant protocol might be insufficient. If the staff do not follow the instructions correctly, but the instructions are adequate, more information and/or time allocated, as well as repeated controls, could possibly improve hygiene standards. The number of contaminated clipper blades did not differ significantly between hospitals, but the fact that the blades from H3 and H2′s small animal department had fewer colonies could signify better protocols, or better adherence to protocols. H2 was part of the pilot study where 90% of the tested blades from the small animal department, and 100% of the blades from the equine department, were contaminated with bacteria [35]. Awareness of this could subsequently have influenced the staff’s behavior when disinfecting the blades. Poor performance of staff could be an important reason for contaminated clipper blades, but it is likely that it is not the only reason, as the frequency of contaminated clipper blades was high in all hospitals, with different staff and instructions.

None of the disinfection routines concerning clipper blades that are currently implemented in the three veterinary referral hospitals resulted in the efficient eradication of bacteria. This carries a risk of spreading opportunistic pathogens to vulnerable patients, which could also include multidrug-resistant bacteria. There was no growth of relevant dermatophytes on the clipper blades.

## 5. Conclusions

There was a high prevalence of bacterial contamination on non-sterilized clipper blades and a significant difference in bacterial contamination between the sterilized and the disinfected clipper blades, regardless of the hospital and which disinfection routine was used. Sterilization is a more reliable way to ensure that clipper blades are clean before they are used on a new patient.

## Data Availability

The data contained within the article.

## Appendix A

Disinfection routines in hospitals H1, H2 (small animals and equine departments), and H3. Translated verbatim to the greatest extent possible by the authors, from instructions in Swedish that were available to staff in the different hospitals.

**H1**

1. Clean the machine of all hair and dirt with the help of a toothbrush.
2. Use an antibacterial wipe to clean the machine.
3. Carefully roll the cord around the machine.
4. The small machines shall be unscrewed and cleaned between patients.

The clipper blades shall be cleaned with a toothbrush and our antibacterial wipes after patient use. After every time that they have been cleaned, the clipper blades should be sprayed with Kruuse clipper spray to disinfect them on the inside, and to blow away hair-or dirt.

Comment from the translator: Antibacterial wipe contains isopropanol (45%)

**H2 Small animal clinic**

Large clippers:

1. Clean by brushing the toothbrush on both the clipper blade and clipper to access small corners.
2. The blade consists of two rows of tines; push the upper one to the side and brush, then push it to the other side and brush.
3. After that, use Ytdes 50 + special (NOT LIFECLEAN), and wipe the machine and clipper blade.
4. Use oil for the blades.
5. Let it dry on the “clean” side.
  Comment: Ytdes 50 + contains isopropanol (40%) and etanol (10%).
  Small clippers:
6. Clean by brushing the toothbrush on both the clipper blade and machine to access small corners.
7. Unscrew the blade from the machine, and brush inside the clipper machine.
8. Put the screw in a designated container.
9. Then, use Ytdes + 50 special (NOT LIFECLEAN) and wipe the machine and clipper blade.
10. Then, use oil for the blades.
11. Let it dry on the “clean”-side, with the small machine upside down.
12. Screw together when dried.

Comment from the translator: Ytdes 50 + contains isopropanol (40%) and ethanol (10%)

**H2 Equine clinic**

1. Pour 2-3 cm BLADE WASH in the bottle’s lid.
2. Run the clipper blade (still attached to the machine) in the solution for 5–10 s, just dip the blade.
3. Detach the blade and let it stay for some minute in the solution.
4. Brush with the tooth brush between all the small areas that are possible. If the dirt doesn’t disappear, dip the clipper blade in BLADE WASH again and leave it for a bit.
5. When has been cleaned with the tooth brush, blow it dry with the device for compressed air.
6. Spray the whole clipper blade with CLIPPERCIDE SPRAY and put it in the designated parking for clipper blades.

Comment from the translator: Clippercide spray contains isopropanol (45.6 %), O-Phenylphenol (0.41 %) and inert ingredients.

**H3**

Small clippers can only be used for shaving before blood tests and catheter placement.

Clean the clipper directly after use.

1. Brush the blade clean from fur with a toothbrush.
2. While the machine is running, flush the blade with surface disinfectant (YTDES). Wipe the clipper and the clipper machine.
3. When the disinfection has evaporated, start the machine and oil the blade. Do not overdose; three drops are enough!
4. Put the clipper with the blade facing downwards on a tray.
5. If you need to charge the machine, make sure that it is clean, and that the disinfectant has evaporated before you place it in the charger.
6. The toothbrush and tray need to be changed every day. Throw the used ones away. Mark the new ones with the date.
7. Clean the clipper blades daily with Bladewash as well.

Comment from the translator: Ytdes contains 40% isopropanol, sometimes one that contains 75% etanol is used too.

## Appendix B

**Table A1:** Bacterial growth on clipper blades from hospital 1.

Clipper Blade	Bacteria	Growth
H1.1	Grampositive cocci Grampositive cocci	Moderate Sparse
H1.2	*Bacillus* species	Rich
H1.3	Grampositive cocci *Psychrobacter sanguinis*	Moderate Moderate
H1.4	Grampositive cocci *Macroccus canis* *Bacillus* species	Rich Moderate Moderate
H1.5	*Bacillus* species (cereus group) Grampositive cocci	Rich Moderate
H1.6	*Bacillus* species *Staphylococcus aureus*	Moderate Moderate
H1.7	*Bacillus* species (cereus group)	Rich
H1.8	*Staphylococcus epidermidis* *Bacillus* species (cereus group) *Micrococcus luteus* *Moraxella* species	Sparse Sparse Sparse Sparse
H1.9	Grampositive cocci Grampositive cocci	Moderate Moderate
H1.10	Grampositive cocci *Macrococcus canis*	Moderate Moderate
H1.11	*Bacillus* species (cereus group) *Rothia endophytica*	Rich Sparse

**Table A2:** Bacterial growth on clipper blades from the small animal department at hospital 2.

Clipper Blade	Bacteria	Growth	After Enrichment
H2.1	*Bacillus subtilis*	Single colonies	
H2.2	No growth		Grampositive cocci
H2.3	*Micrococcus luteus*	Single colonies	*Staphylococcus epidermidis*
H2.4	No growth		*Staphylococcus saprophyticus* *Staphylococcus hominis*
H2.5	*Staphylococcus warneri* *Staphylococcus hominis*	Single colonies Single colonies	
H2.6	*Staphylococcus pseudintermedius* *Leclercia adecarboxylata* *Staphylococcus hominis* Grampositive cocci	Moderate Single colonies Sparse Sparse	
H2.7	*Bacillus* species (cereus group) *Staphylococcus pseudintermedius*	Single colonies Single colonies	
H2.8	*Staphylococcus warneri* *Staphylococcus* species	Single colonies Single colonies	
H2.9	No growth		*Bacillus pumilus*
H2.10	No growth		*Bacillus* species (cereus group)
H2.11	Gramnegative rods	Single colonies	

**Table A3:** Bacterial growth on clipper blades from the equine department at hospital 2.

Clipper Blade	Bacteria	Growth
H2.E1	Grampositive cocci *Acinetobacter lwoffii* *Psychrobacter* species	Sparse Sparse Sparse
H2.E2	*Macrococcus brunesis* Grampositive cocci	Moderate Moderate
H2.E3	*Acinetobacter radioresistant* *Acinetobacter lwoffi* *Micrococcus* species	Sparse Sparse Single colonies
H2.E4	Grampositive cocci *Bacillus* species	Single colonies Single colonies
H2.E5	*Staphylococcus vitulinus* *Aerococcus viridans* Grampositive rods Grampositive rods	Sparse Sparse Sparse Single colonies
H2.E6	Grampositive cocci *Acinetobacter pseudolwoffii* *Aerococcus viridans* *Bacillus* species	Sparse Sparse Sparse Sparse
H2.E7	Gramnegative rods *Aerococcus viridans* *Corynebacterium* species	Moderate Sparse Moderate
H2.E8	*Staphylococcus simulans* *Bacillus pumilus* *Staphylococcus equorum* Gramnegative rods	Sparse Sparse Sparse Single colonies
H2.E9	*Bacillus licheniformis* *Bacillus* species (cereus group)	Single colonies Single colonies
H2.E10	*Staphylococcus equorum* *Aerococcus viridans* Grampositive cocci Gramnegative rods	Sparse Sparse Sparse Single colonies
H2.E11	Grampositive cocci *Staphylococcus saprophyticus* Gramnegative rods Grampositive cocci	Sparse Sparse Sparse Single colonies

**Table A4:** Bacterial growth on clipper blades from hospital 3.

Clipper Blade	Bacteria	Growth	After Enrichment
H3.1	Grampositive diplococci Grampositive cocci	Rich Moderate	
H3.2	*Moraxella osloensis* Grampositive cocci *Staphylococcus pseudintermedius*	Sparse Sparse Sparse	
H3.3	*Micrococcus luteus* *Staphylococcus epidermidis*	Single colonies Single colonies	
H3.4	No growth		No growth
H3.5	Grampositive cocci Gramnegative rods Grampositive cocci *Staphylococcus petrasii*	Sparse Sparse Sparse Sparse	
H3.6	No growth		No growth
H3.7	*Staphylococcus capitis*	Single colonies	
H3.8	No growth		No growth
H3.9	Grampositive cocci *Moraxella* species	Sparse Single colonies	
H3.10	*Enterococcus faecium*	Single colonies	
H3.11	No growth		*Bacillus* species

**Table A5:** Bacterial growth on sterilized clipper blades.

Clipper Blade	Bacteria	Growth
AUT1	No growth	
AUT2	No growth	
AUT3	No growth	
AUT4	No growth	
AUT5	No growth	
